# Increase in longevity and amelioration of pesticide toxicity by natural levels of dietary phytochemicals in the honey bee, *Apis mellifera*

**DOI:** 10.1371/journal.pone.0243364

**Published:** 2020-12-09

**Authors:** Ling-Hsiu Liao, Daniel J. Pearlstein, Wen-Yen Wu, Allison G. Kelley, William M. Montag, Edward M. Hsieh, May R. Berenbaum

**Affiliations:** 1 Department of Entomology, University of Illinois at Urbana-Champaign, Urbana, Illinois, United States of America; 2 Parkland College, Champaign, IL, United States of America; Ghent University, BELGIUM

## Abstract

For the past decade, migratory beekeepers who provide honey bees for pollination services have experienced substantial colony losses on a recurring basis that have been attributed in part to exposure to insecticides, fungicides, or their combinations applied to crops. The phytochemicals *p*-coumaric acid and quercetin, which occur naturally in a wide variety of bee foods, including beebread and many types of honey, can enhance adult bee longevity and reduce the toxicity of certain pesticides. How variation in concentrations of natural dietary constituents affects interactions with xenobiotics, including synthetic pesticides, encountered in agroecosystems remains an open question. We tested the effects of these two phytochemicals at a range of natural concentrations on impacts of consuming propiconazole and chlorantraniliprole, a triazole fungicide and an insecticide frequently applied as a tank mix to almond trees during bloom in California’s Central Valley. Propiconazole, even at low field concentrations, significantly reduced survival and longevity when consumed by adult bees in a sugar-based diet. The effects of propiconazole in combination with chlorantraniliprole enhanced mortality risk. The detrimental effects of the two pesticides were for the most part reduced when either or both of the phytochemicals were present in the diet. These findings suggest that honey bees may depend on non-nutritive but physiologically active phytochemical components of their natural foods for ameliorating xenobiotic stress, although only over a certain range of concentrations; particularly at the high end of the natural range, certain combinations can incur additive toxicity. Thus, efforts to develop nectar or pollen substitutes with phytochemicals to boost insecticide tolerance or immunity or to evaluate toxicity of pesticides to pollinators should take concentration-dependent effects of phytochemicals into consideration.

## Introduction

Many fungal diseases initiate their disease cycles during plant host flowering. For management of these fungal pathogens, many orchard crops are treated with fungicides during bloom [1], when honey bees (*Apis mellifera*), the principal managed pollinators in U.S. agriculture, are brought in to provide pollination services. Fungicides thus are among the pesticides most frequently found as contaminants in hives [2]. Even though most fungicides are considered “bee-safe”, low levels of exposure can cause sublethal health effects that lead to weaker colonies or even colony losses, reducing pollination efficiency and decreasing yields [3]. The intermittency of honey bee losses experienced by beekeepers after delivery of pollination services in California’s Central Valley, however, suggests factors other than fungicides alone influence the magnitude of their impact on bees. Fungicides are generally ingested by bees along with dietary phytochemicals present naturally in nectar and pollen as well as with insecticides applied by growers for pest management; these chemicals can all interact to influence bee health. In fact, more than 95% of insecticide applications to almond orchards are made concurrently with fungicide applications [4].

In general, honey bees use nectar from a variety of plant species as the foundation for making honey, a supplementary food for worker and drone grubs and an energy source for adult workers [5]. Honeys thus contain a diversity of phytochemicals, the identities of which depend on floral sources. Among the most commonly occurring phytochemicals in nectar are phenolic acids and flavonols, which are also found in pollen, the principal source of dietary protein, as well as propolis, a material manufactured by bees from plant resins mixed with salivary secretions and beeswax and used as an antimicrobial hive sealant [6]. Composition and concentrations of phenolic acids and flavonols in floral resources, and thus in bee foods, can vary both inter- and intra-specifically [e.g., 7], depending on factors such as genetics [e.g., 8], geography [e.g., 9] and agricultural management [e.g., 10,11].

Among phenolic acids and flavonols, *p*-coumaric acid and quercetin respectively stand out in having demonstrable impacts on honey bee physiology and health. When consumed by adult bees, they enhance longevity [12,13]. Moreover, the presence of *p-*coumaric acid in the diet can reduce spore-load in adult bees infected with the fungal pathogen *Nosema ceranae* [13], which may be due to its ability to upregulate genes encoding antimicrobial peptides [14]. Both *p*-coumaric acid and quercetin increased the survival rate of bees when ingested along with the pyrethroids bifenthrin and β-cyfluthrin [12], the in-hive acaricide coumaphos [15], or the neonicotinoid imidacloprid [16]. The protective effect of *p*-coumaric acid and quercetin against dietary toxins in adult bees is likely due to the ability of both compounds to upregulate specific detoxification genes in both larval and adult stages, particularly those in the CYP9Q subfamily encoding enzymes that metabolize pesticides.

In a study examining impacts of commonly used insecticides and fungicides during almond bloom on honey bee health, Wade et al. [4] determined that the combination of the insecticide chlorantraniliprole and the triazole fungicide propiconazole was seven times more toxic to adult bees than chlorantraniliprole alone. Chlorantraniliprole was the first commercialized member of the anthranilic diamide class of insecticides, which target insect ryanodine receptors, a class of intracellular calcium channels [17]. Altacor®, one of chlorantraniliprole’s many formulated products, is applied on almonds for control of peach twig borer (*Anarsia lineatella*) [18]. Propiconazole is a broad-spectrum triazole fungicide used for management of brown rot blossom blight (*Monilinia laxa*) and various pathogenic fungi in almonds. Like chlorantraniliprole, it is frequently applied during bloom. These two pesticides thus are often combined and applied as a tank mix in almond orchards from bud break to bloom [19].

Fungicides in the triazole class have long been known to act as synergists that enhance toxicity of insecticides to honey bees [reviewed in 4]. Collectively known as ergosterol-biosynthesis inhibitor (EBI) fungicides, they owe their fungicidal activity to their ability to interfere with the CYP51 (sterol 14-alpha-demethylase) enzyme involved in ergosterol synthesis in pathogenic fungi. Molecular modeling and *in silico* docking studies of honey bee CYP9Q1 have shown that propiconazole and other triazole fungicides dock in the active pocket of the catalytic site and thus are potential competitive inhibitors of insecticide detoxification [20]. Such triazole-mediated inhibition of honey bee P450-mediated insecticide detoxification likely accounts for the synergistic enhancement by these fungicides of insecticide toxicity.

Because both *p*-coumaric acid and quercetin upregulate CYP9Q P450s, they could potentially ameliorate synergistic interactions between insecticides and fungicides if ingested concurrently with these pesticides. However, the limited capacity of honey bees to manage detoxification of combinations of chemicals, possibly associated with their reduced inventory of detoxification P450s [21], suggests that phytochemicals may also interfere with xenobiotic detoxification. In fact, consumption of high concentrations of quercetin by adult bees can have adverse impacts on their behavior [22] and physiology [23]. In order to determine whether the optimal range of concentration for amelioration of xenobiotic toxicity by honey phytochemicals corresponds to the typical range of concentrations naturally encountered by adult bees, we first surveyed the literature to document the range of reported concentrations of *p*-coumaric acid and quercetin in nectar, honey, pollen and beebread. We then examined the effects of consuming these two phytochemicals, alone and in combination, across the reported range of natural concentrations on adult bee longevity in the presence and absence of chlorantraniliprole and propiconazole in their diet.

## Materials and methods

### Literature review

We conducted a survey of reported ranges of phytochemical concentrations searching PubMed and Google Scholar databases with the search terms “quercetin” or “*p*-coumaric acid” combined with either “honey”, “pollen”, “beebread”, “propolis” or “royal jelly” and “concentration” for papers published up to and including 2015. The reported phytochemical concentrations (means and/or ranges), type of bee products, botanical origin, and geographic sources of samples from previous literature in the papers produced by the search were recorded. If phytochemical concentrations were reported in a paper as a range, the maximum value of each sample was recorded on a separate column (Max). The reports with no quantitative phytochemical data or with only relative concentrations were not included in our final statistical analyses and tables. When the reported phytochemical concentration was lower than the detection limit of their method, we considered it as zero. All units of concentration in literature were converted into μM for cross-comparison. Pivot tables based on the bee product, botanical source, and geographic source were created in Excel (Version 16.21.1; Microsoft Corp., Redmond, WA) to sort and summarize the results of natural phytochemical concentrations of in forms of descriptive statistics.

### Chemicals

Commercial granulated cane sugar (Domino Foods, Inc., West Palm Beach, FL, and Great Value, Walmart Inc., Bentonville, AR) was purchased at local grocery stores. Dimethyl sulfoxide (DMSO; D128) was obtained from Fisher Scientific International, Inc., Pittsburgh, PA, USA. All other chemicals were purchased from Sigma Aldrich, St. Louis, MI, USA, including casein (C3400), chlorantraniliprole (32510), propiconazole (45642), *p*-coumaric acid (C9008), and quercetin (Q4951).

### General protocol for longevity bioassays

To examine diet effects on longevity, we followed the general protocols and the basic diet (comprising 50% sugar water, 0.25% DMSO, with casein added to bring the protein/carbohydrate ratio to 1:12) used in previous studies [12,16]. Colonies in apiaries maintained by the University of Illinois at Urbana-Champaign in Urbana, IL, were used as sources of bees during the fall of 2016 (the first pesticide-phytochemical interaction assay) and summer of 2017 (all remaining assays). All colonies providing bees for bioassays were strong and showed no signs of disease (with either no mites or very low mite counts on nurse bees, healthy brood patterns and no sign of dysentery around the entrance of hive) when we pulled the brood frames. For each bioassay, brood frames were obtained from three unrelated hives and kept in an incubator (34°C, 50% relative humidity). Emerging one-day-old adult bees were collected daily. Each replicate (with all concentrations of phytochemicals and pesticides) was carried out with one-day-old bees from each of the three colonies on the same day. Three to four replicates per each hive of three hives were carried out in all experiments within a week, with one exception; due to the seasonal constraints, the first pesticide-phytochemical interaction assay (30 ppm propiconazole and/or 2ppm chlorantraniliprole), carried out in October 2016, comprised six replicates with one-day-old adults pooled from three unrelated hives. Observation cages were constructed and provisioned with a basic defined diet differentially amended with test compounds to assess impacts on longevity. The assays were carried out in 9 oz. (266 ml) observation cages (made from disposable PET plastic cups, catalogue #:500CC9, WebstaurantStore, Lancaster, PA) containing 25 bees; each cage contained one 2-ml microcentrifuge tube with fresh sugar water diet (changed daily) and one with distilled water (changed weekly) for access *ad libitum*.

### Effective range of phytochemicals on adult honey bee survival

Two phytochemicals in four concentrations, *p*-coumaric acid (5, 50, 500, 1000 μM) and quercetin (12.5, 25, 250, 1000 μM), based on levels in natural concentrations in honey, pollen, or propolis (Table 1), were tested, along with a phytochemical-free control, in order to identify phytochemical concentrations with beneficial effects on honey bees. Mortality of bees within each cage was recorded in 8-hr intervals (8:00 am, 4:00 pm, 12:00 am) for approximately one week.

**Table 1:** Naturally occurring concentrations of quercetin and *p*-coumaric acid in bee products^1^.

		mean concentration (μM)^2^			Max^3^ (μM)	Ref.
		Min	Average	Max		
Quercetin	royal jelly		0.7			[28]
	Honey	0.0	26.0	228.3	n/a	[9,28–44]
	Pollen	5.8	1 190.8	3 250.0	n/a	[45–51]
	Propolis	0.0	4 227.5	27 193.6	54 648.7^4^	[28,52–61]
*p*-coumaric acid	Honey	0.0	42.5	1 914.1	n/a	[29–31,35,36,41–43,62–64]
	Pollen	4.9	680.8	2 499.3	n/a	[45,46,50,51,65]
	Propolis	0.0	14 413.9	69 492.2	82 293.3^5^	[52,53,55,57–59,66–75]

^1^ Upper and lower limits of each group of bee products overlap each other. Phytochemical concentrations varied according to botanical origin (S1 Table) and geographic source (S2 Table).

^2^ The data, originally reported as a mean value in the cited paper, reported here.

^3^ This column shows the maximum value of the concentration of the phytochemical reported in the cited paper as a range. Studies in which values were reported as lower than the maximum mean concentration (sleft-hand column) were entered here as “n/a”.

^4^ Aliyazıcıoglu et al. [52] show this maximum value of quercetin in propolis.

^5^ Bertelli et al. [53] show this maximum value of *p*-coumaric acid in propolis.

### Pesticide-phytochemical interaction assay

The two phytochemicals (*p*-coumaric acid and quercetin) in four combinations (control, 500 μM p-coumaric acid, 250 μM quercetin and 500 μM *p*-coumaric acid + 250 μM quercetin) and three pesticide combinations (2 ppm chlorantraniliprole, 30 ppm propiconazole, and 2 ppm chlorantraniliprole + 30 ppm propiconazole) were tested. The concentration of chlorantraniliprole used in this experiment was based on previous reports of maximum field residues [24] and the concentration of propiconazole was based on the assumed daily dosage with its LD_50_ [25] over the average lifespan and the average daily sugar water consumption per caged bee (25 μl as determined previously [12]).

The assay aimed at comparing a range of pesticide concentrations was performed in late summer 2016. Due to seasonal constraints, six replicates were carried out with a three-hive-mixed population of bees emerging within a four-day period. To obtain sufficient numbers of individuals for all replicates for each day of testing and minimize the genomic variation caused by hive identity, equivalent quantities (by weight) of one-day-old bees from each colony were intermingled in a container and then assigned randomly in lots of 25 to cages, the same group size used in earlier assays. The number of surviving bees in each cage was recorded daily until overall mortality reached 80% (about 30 days). In previous longevity assays we have conducted with honey bees [12,16], survival curves with endpoints of 80% mortality revealed treatment effects; accordingly, we used the endpoint of 80% mortality for this experiment.

### Propiconazole: Chlorantraniliprole 9:4 tank-mix ratio assay

We chose for bioassay the ratio 9:4 propiconazole: chlorantraniliprole suggested for field tank-mixed applications in almond orchards and examined by Wade et al. [4] in larval bioassays and adult topical toxicity assays. This ratio was tested at two concentrations: 0.9 ppm propiconazole + 0.4 ppm chlorantraniliprole and 90 ppm propiconazole + 40 ppm chlorantraniliprole. These concentrations were previously determined to be sublethal by Wade et al. [4]. For the first ten days of the assay, survivorship was evaluated every 8 hours; for the remainder of the assay, survivorship was monitored once daily until overall mortality reached 80% (about 10 days on diets with high pesticide concentrations and 25 days on diets with low pesticide concentrations).

Diets containing *p*-coumaric acid (5, 50, 500, 1000 μM) and quercetin (12.5, 25, 250, 1000 μM) as well as a phytochemical-free control diet were tested as described earlier with chlorantraniliprole and propiconazole individually and in combination at two concentrations (low concentration: 0.9 ppm propiconazole, 0.4 ppm chlorantraniliprole, and 0.9 ppm propiconazole + 0.4 ppm chlorantraniliprole, high concentration 90 ppm propiconazole + 40 ppm chlorantraniliprole). We tested 36 combination diets in total. For ten days, mortality was recorded at 8-hour intervals; for the remainder of the assay, mortality was recorded once daily until overall mortality reached 80% (about 10 days on diets with high pesticide concentrations and 25 days on diets with low pesticide concentrations).

### Statistical analysis of bioassays

Statistical analyses were conducted using SPSS software (version 22.0; IBM Corp., Armonk, NY, USA) and OriginPro 2016 software (OriginLab Corporation, Northampton, MA, USA). OriginPro was used to plot Kaplan-Meier survival curves with the estimated means, and the medians with the Kaplan-Meier estimator. Significant differences between treatment and control were determined through the Tarone-Ware test [26]. The hazard of death according to dietary phytochemicals and pesticide concentrations was evaluated with Cox's proportional hazards regression models [27]. Data were adjusted for hive identity as a covariate stratum if available.

## Results

### Effective range of phytochemicals on adult honey bee survival

In general, across bee products, quercetin and *p*-coumaric acid concentrations decreased in abundance in the order propolis > pollen > honey > royal jelly (Table 1), with the highest mean concentration of quercetin and *p*-coumaric acid concentrations in propolis averaging 4 227.5 ± 2 214.5 and 14 413.9 ± 6 433.6 μM, respectively, and with the lowest quercetin concentration at trace levels (0.7 μM) in royal jelly (which lacked even traces of *p*-coumaric acid). Mean concentrations of quercetin (26.0 ± 4.7 μM) and *p*-coumaric acid (42.5 ± 27.2 μM) in honey were lower than mean concentrations in pollen, with quercetin at 1 190.8 ± 687.4 μM and *p*-coumaric acid at 680.8 ± 606.9 μM.

**Fig 1:**
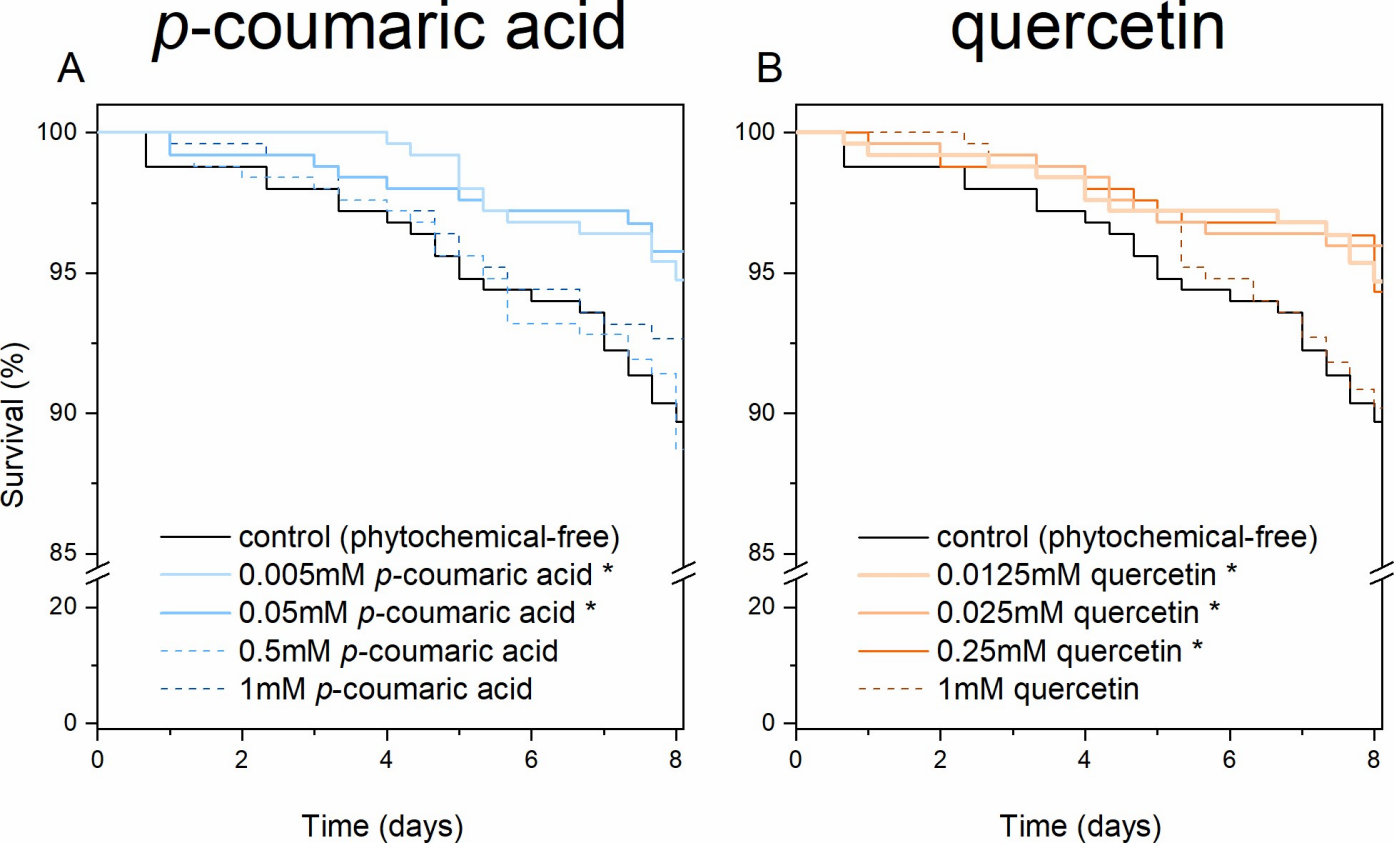
Kaplan–Meier plots of honey bee longevity with dietary amendments at a range of concentrations. (A) *p*-coumaric acid and (B) quercetin. (n = 1 250 for each phytochemical group and n = 250 for each phytochemical concentration subgroup, solid line and * = *p* < 0.05, Tarone-Ware test between the treatment and control).

Consuming *p*-coumaric acid at 0.005 mM (*χ*^2^ = 4.49, *p* = 0.03; Cox model, *p* = 0.04, hazard ratio (HR): 0.48) and 0.05 mM (*χ*^2^ = 6.68, *p* = 0.01; Cox model, *p* = 0.01, HR: 0.40; Fig 1A) results in enhanced survival rates of adult bees, as does quercetin at 0.0125 mM (*χ*^2^ = 4.45, *p* = 0.04; Cox model, *p* = 0.04, HR: 0.48), 0.025 mM (*χ*^2^ = 6.49, *p* = 0.01; Cox model, *p* = 0.01, HR: 0.40) and 0.25 mM (*χ*^2^ = 4.55, *p* = 0.03; Cox model, *p* = 0.04, HR: 0.48; Fig 1B). The effective range of both phytochemicals reduced the hazard ratio (HR) by approximately 60% and encompassed the natural range concentrations in honey extending into the lower end of the range of concentrations in pollen.

### Pesticide-phytochemical interaction assay

Overall, according to the Cox regression model, the diet containing pesticides, 2- ppm chlorantraniliprole and 30 ppm propiconazole, decreased the probability of survival (HR = 1.37, *p* < 0.001, propiconazole; HR = 1.21, *p* < 0.01, chlorantraniliprole); and they also showed a synergistic effect (HR = 1.37, *p* < 0.001, combination vs chlorantraniliprole; S1 Fig). Consumption of phytochemicals influenced survival probabilities; diets containing *p*-coumaric acid reduced mortality risk whereas diets containing quercetin enhanced mortality risk compared to the phytochemical-free control diet (HR = 0.87 and 1.17, *p* < 0.05, respectively). The negative effects of quercetin were due to its interaction with chlorantraniliprole and propiconazole in these specific concentrations.

**Fig 2:**
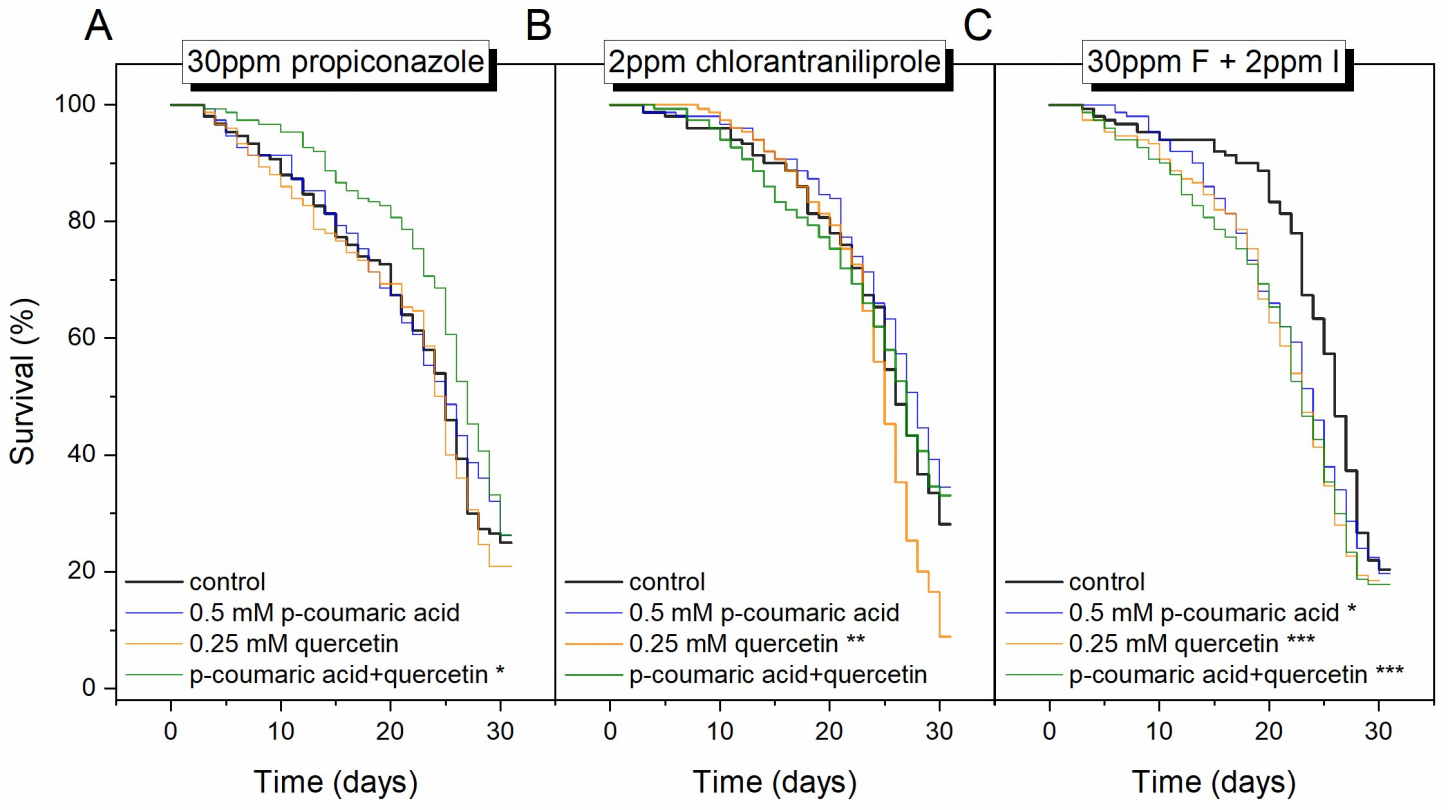
Kaplan–Meier plots of honey bee longevity on diets varying in phytochemical and pesticide content. (A) 30 ppm fungicide propiconazole (B) 2 ppm insecticide chlorantraniliprole, (C) 30 ppm propiconazole + 2 ppm chlorantraniliprole (F: fungicide propiconazole, I: insecticide chlorantraniliprole; n = 600 for each pesticide group and n = 150 for each phytochemical subgroup; *** = *p* < 0.001, ** = *p* < 0.01, * = *p* < 0.05, Tarone-Ware test between the treatment and control).

Based on the Kaplan–Meier survival analysis, ingesting 0.25 mM quercetin + 0.5 mM *p*-coumaric improved tolerance of propiconazole in the diet at 30 ppm (*χ*^2^ = 6.49, *p* = 0.01, Tarone-Ware test; Fig 2A); however, when these two phytochemicals were ingested individually with 30 ppm propiconazole, the survival curve was unaffected. Phytochemicals also did not improve survival on diets containing 2 ppm chlorantraniliprole. Quercetin with 2 ppm chlorantraniliprole even show a negative effect (χ^2^ = 6.97, *p* < 0.01, Tarone-Ware test; Fig 2B). Moreover, on the diet amended with 2 ppm chlorantraniliprole + 30 ppm propiconazole, consuming phytochemicals either individually or together significantly increased worker mortality (χ^2^ = 6.12, 13.39 and 12.95, *p* < 0.05, *p*-coumaric acid, quercetin, and combine respectively, Tarone-Ware test; Fig 2C).

### Chlorantraniliprole and propiconazole tank-mix 9:4 ratio assay

**Fig 3:**
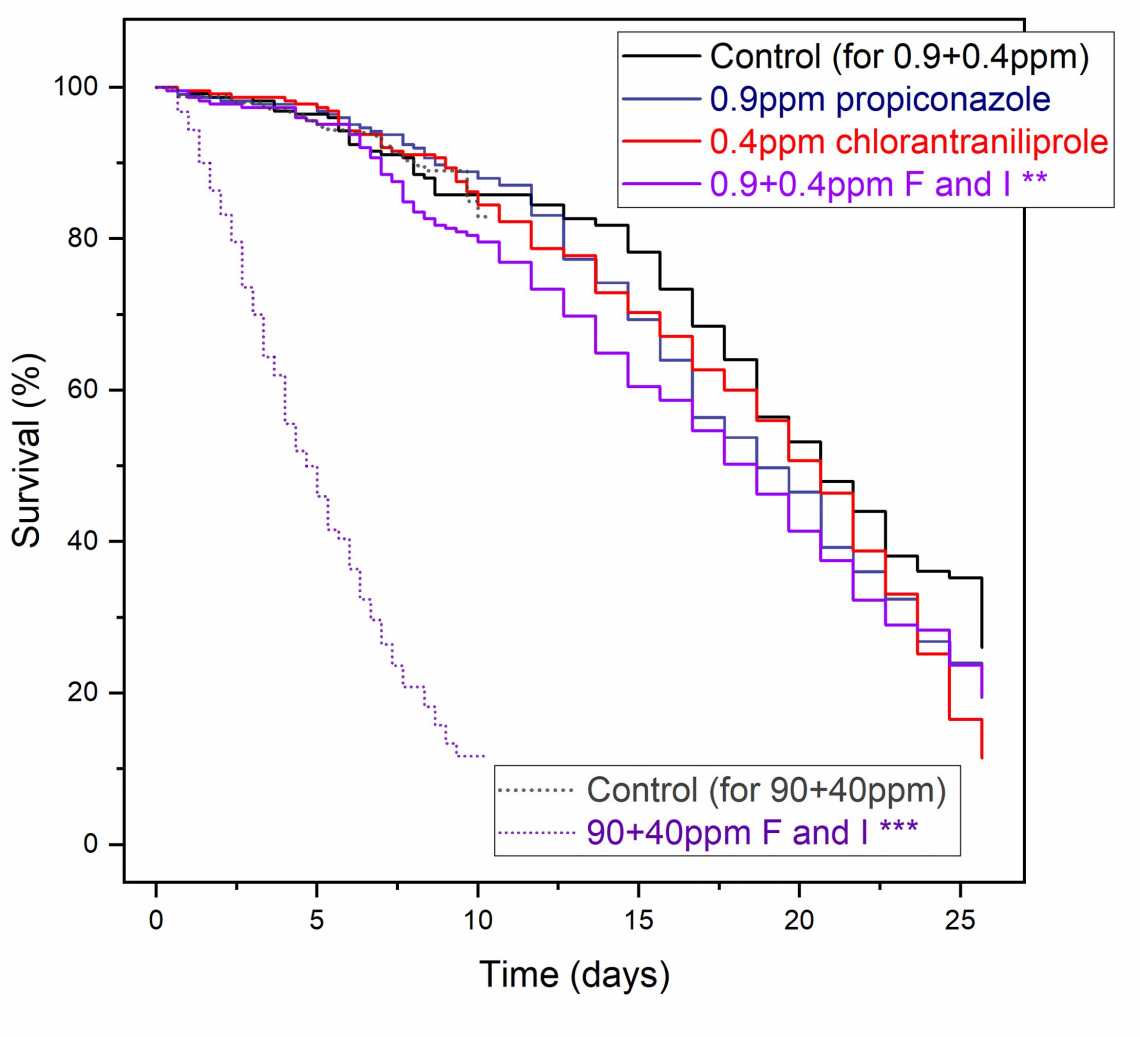
Kaplan–Meier survival curves of honey bees on tank-mix ratio pesticide treatments. Honey bees were fed with low [0.9 ppm propiconazole (blue solid line), 0.4 ppm chlorantraniliprole (red solid line), and 0.9 ppm propiconazole + 0.4ppm chlorantraniliprole (purple solid line)] or high [90 ppm propiconazole + 40 ppm chlorantraniliprole(purple dashed line)] concentrations of tank-mixed pesticides. Bees consuming combinations of pesticides experienced reduced longevity relative to bees consuming unamended control diet and relative to bees consuming chlorantraniliprole or propiconazole alone (F: fungicide propiconazole, I: insecticide chlorantraniliprole; n = 900 in 0.9+0.4 ppm and 500 in 90+40ppm subgroup, *** = *p* < 0.001, ** = *p* < 0.01, Tarone-Ware test between the treatment and control).

Tank-mixed fungicide and insecticide (propiconazole + chlorantraniliprole) at both low (0.9+0.4 ppm) and high (90+40 ppm) concentrations decreased survival (*χ*^2^ = 9.23 and 262.46, *p* = 0.002 and < 0.001, respectively, Tarone-Ware test, Fig 3). The high-concentration combination of fungicide and insecticide increased the risk of death 14-fold (HR: 14.06, *p* < 0.001, Cox model) compared to control; the low-concentration combination increased the risk of death by 40% (HR: 1.40, *p =* 0.003 <0.01, Cox model), while the risk of consuming propiconazole or chlorantraniliprole alone at these low concentrations was not significantly different from the control (*p* > 0.05, Kaplan–Meier and Cox model).

**Fig 4:**
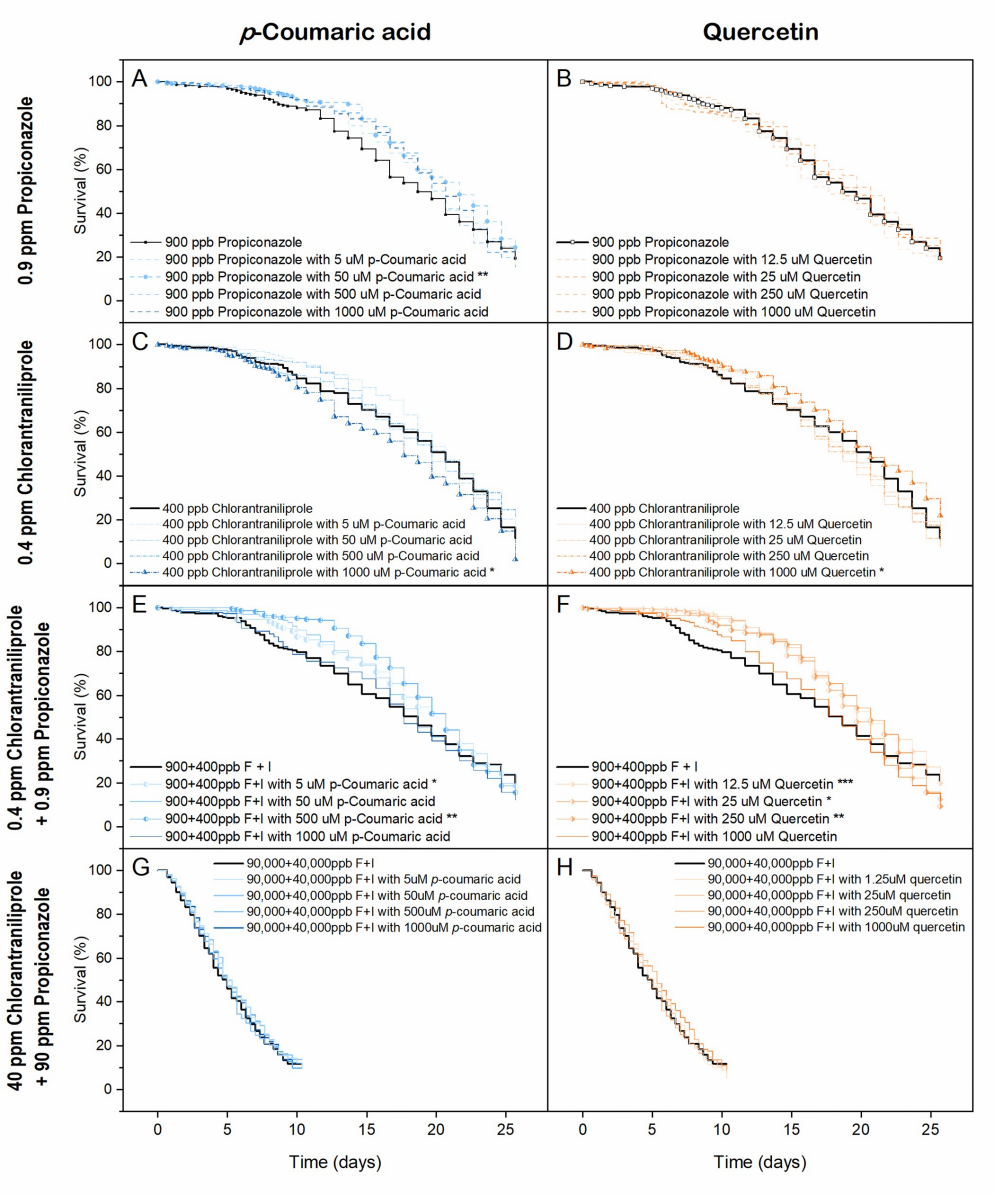
Kaplan–Meier survival curves of honey bees on dietary tank-mix pesticides and phytochemicals. Diet treatments included: (A) 0.9 ppm propiconazole amendment with *p-*coumaric acid at a range of concentrations, (B) 0.9 ppm propiconazole amendment with quercetin at a range of concentrations, (C) 0.4 ppm chlorantraniliprole amendment with *p-*coumaric acid at a range of concentrations, (E) 0.9 ppm propiconazole + 0.4 ppm chlorantraniliprole amendment + *p-*coumaric acid at a range of concentrations, (F) 0.9 ppm propiconazole + 0.4 ppm chlorantraniliprole amendment + with quercetin at a range of concentrations, (G) 90 ppm propiconazole + 40 ppm chlorantraniliprole amendment with *p-*coumaric acid at a range of concentrations, and (H) 90 ppm propiconazole + 40 ppm chlorantraniliprole amendment with *p-*coumaric acid at a range of concentrations (F: fungicide propiconazole, I: insecticide chlorantraniliprole; n = 1 125 for each pesticide group in Fig 4A-4F and n = 1 250 for each pesticide group in Fig 4G and 4H; *** = *p* < 0.001, ** = *p* < 0.01, * = *p* < 0.05, Tarone-Ware test).

When consumed in diets containing low concentrations of pesticides (0.9 ppm propiconazole and 0.4 ppm chlorantraniliprole; Fig 4A–4F), lifespan extension occurred on the 50 μM *p*-coumaric acid + 0.9 ppm propiconazole diets (*χ*^2^ = 9.54, *p =* 0.002, Tarone-Ware test; Fig 4A) and 1000 μM quercetin + 0.4 ppm chlorantraniliprole diets (*χ*^2^ = 4.24, *p =* 0.04, Tarone-Ware test; Fig 4D). Similarly, consumption of either *p*-coumaric acid at 5 or 500 μM (*χ*^2^ = 5.55 and 7.97, *p =* 0.02 and 0.005, Tarone-Ware test; Fig 4E) or quercetin at 12.5, 25 or 250 μM (*χ*^2^ = 12.00, 3.98 and 8.28, *p* < 0.001, <0.05 and <0.01, respectively, Tarone-Ware test; Fig 4F) decreased the toxicity of the 0.9 ppm propiconazole + 0.4 ppm chlorantraniliprole diet, extending mean lifespan by 9–15% (39.3–60.3 h) and 12–16%, (49.5–68.1 h), respectively. Overall, *p*-coumaric acid at concentrations of 5, 50, and 500 μM decreased the risk of death by 12%-13% (HR: 0.87–0.88, *p* < 0.05, Cox model) compared to the phytochemical-free diet.

Phytochemical amelioration of toxicity, however, did not occur when pesticides were present at high concentrations. Consumption of diets containing 90 ppm propiconazole + 40 ppm chlorantraniliprole reduced lifespan significantly (Fig 3), but phytochemical consumption did not affect this outcome (Fig 4G and 4H). Additionally, a synergistic effect was observed between *p*-coumaric acid and 0.4 ppm chlorantraniliprole, whereby diets containing 1 000 μM *p*-coumaric acid and chlorantraniliprole reduced survival relative to diets containing the insecticide alone (*χ*^2^ = 6.47, *p =* 0.01, Tarone-Ware test; Fig 4C), causing a 7% reduction in lifespan (31 hours) compared to the control group.

## Discussion

The beneficial effects of two routinely ingested phytochemicals, *p*-coumaric acid and quercetin, on adult honey bees appear upon consumption of concentrations naturally encountered in floral foods by adult bees over their lifetimes, suggesting that the detoxification system of *A*. *mellifera* reflects evolutionary specialization for a phenolic-rich diet. We determined the range of concentrations of phytochemicals most effective at extending survival in the presence and absence of pesticides. In the absence of pesticides, we found that the phytochemical concentrations tested that correspond to those found in many honeys and some pollens (5–50 μM *p*-coumaric acid and 12.5–250 μM quercetin) (Table 1) significantly promoted bee survival. Similarly, phytochemicals within this range could, depending on the pesticide and its concentration, ameliorate adverse effects. At the highest concentrations tested, which exceed the natural levels in nectar and honey but which are within the range found in pollen and propolis, phytochemicals could exacerbate toxicity in some circumstances. Because propolis is ubiquitous on surfaces throughout the hive, its constituents might be ingested by honey bees via a number of routes—e.g. as a consequence of absorption of its constituents into honey and subsequent ingestion and of social grooming—but propolis itself is not a food and therefore it is unlikely that adult bees ingest quantities of phytochemicals in proportion to their abundance in propolis.

In addition, these two phytochemicals can not only increase the longevity of bees consuming a sugar-based diet, as previously demonstrated, but also enhance survival by alleviating the adverse impacts of fungicides and insecticides frequently found in tank mixes, such as propiconazole and chlorantraniliprole, in a concentration-dependent manner. These two pesticides interact synergistically, most likely due to the P450-inhibiting effects of the triazole fungicide; the related fungicide myclobutanil, e.g., inhibits P450-mediated detoxification of the phytochemical quercetin [20]. Although the mechanism of detoxification of chlorantraniliprole has not yet been determined, cytochrome P450s have been implicated in its detoxification in other insects, such as the diamondback moth *Plutella xylostella* [76]. In examining transcriptional responses of honey bees to dietary pesticide exposure, Christen and Fent [77] reported that consumption of chlorantraniliprole led to upregulation of *CYP9Q2* after 24 hours, which is suggestive of P450-involvement in its metabolism, but these investigators also demonstrated down-regulation of *CYP9Q1* at all concentrations tested and *CYP9Q3* after 48 hours at concentrations of 10 and 100 ng/bee.

The ability of *p*-coumaric acid and quercetin to ameliorate the synergistically enhanced effects of the tank-mixed pesticides in this study is consistent with rescue through enhancement of P450-mediated metabolism. Both of these phytochemicals, abundantly represented in the natural diet of the honey bee, upregulate expression of a diversity of cytochrome P450 genes [20], including all three CYP9Q genes in honey bees, which are involved in the metabolism of many pesticides, including pyrethroids, organophosphates [78] and neonicotinoids [21]. Moreover, the triazole fungicides interact synergistically with neonicotinoids [79,80] and pyrethroids [81] in *A*. *mellifera*. Finally, Mao et al. [20] demonstrated that a number of triazole fungicides, include propiconazole, dock in the CYP9Q catalytic site. These results collectively suggest an important role of CYP450s in honey bee detoxification of propiconazole. Upregulation of these cytochrome P450 genes by phytochemicals may thus compensate, at least in part, for the inhibitory impacts of the triazole fungicides and possibly the anthranilic diamide insecticide chlorantraniliprole.

Other properties of quercetin and *p*-coumaric acid may also contribute to reducing insecticide toxicity and prolonging survival. Quercetin may ameliorate the effects of chlorantraniliprole via inhibiting Ca^2+^-ATPase activity and thereby altering calcium ion fluxes [82–84] or via modulating ryanodine receptors [85,86]. Because chlorantraniliprole [87,88] and propiconazole [89] are both known to induce oxidative stress in other insects, phytochemicals may enhance longevity by attenuating pesticide-induced stress [90]. Quercetin [91,92] and *p*-coumaric acid [93–95] are both known for their antioxidant properties, which may also contribute to a mechanism by which they enhances survival in the presence of pesticides.

Flavonols and phenolic acids are collectively universal constituents of pollens, all of which contain the flavonol quercetin and/or kaempferol as a pollen tube germination signaling compound [96] and screening pigment against ultraviolet light [97–99] and *p*-coumaric acid as the monomeric subunit of the sporopollenin exine capsules [100–103]. Similarly, quercetin is ubiquitous in nectars utilized by honey bees; *p*-coumaric acid is present in many honeys, likely due to their content of pollen grains [14] and has been used as a marker for identifying floral sources in certain honeys [41,104]. Their reliable association with bee food may account for their apparent acquisition of a regulatory function in detoxification of ingested substances. The fact that the ability of these two phytochemicals to extend lifespan and alleviate pesticide toxicity is concentration-dependent should be taken into account in any efforts to incorporate them as additives to food substitutes to improve their quality for beekeeping applications and pollinator health.

## Supporting information

S1 Fig Hazard function of honey bee on diets varying in pesticide content [30 ppm fungicide propiconazole (Fu), 2 ppm insecticide chlorantraniliprole (In), 30 ppm propiconazole + 2 ppm chlorantraniliprole (IF)]. (Cox regression model).
(DOCX)

S1 Table Naturally occurring concentrations of quercetin and *p*-coumaric acid in bee products from different botanical origin. (DOCX)

S2 Table Naturally occurring concentrations of quercetin and *p*-coumaric acid in bee products from different geographic origin. (DOCX)
