# Supplementary material for: Increase in longevity and amelioration of pesticide toxicity by natural levels of dietary phytochemicals in the honey bee, Apis mellifera
Source: PLoS One. 2020 Dec 9;15(12):e0243364. doi: 10.1371/journal.pone.0243364 (PMC7725320; doi:10.1371/journal.pone.0243364)
Supplement: S1 Fig — (Cox regression model). (DOCX) [file pone.0243364.s001.docx]

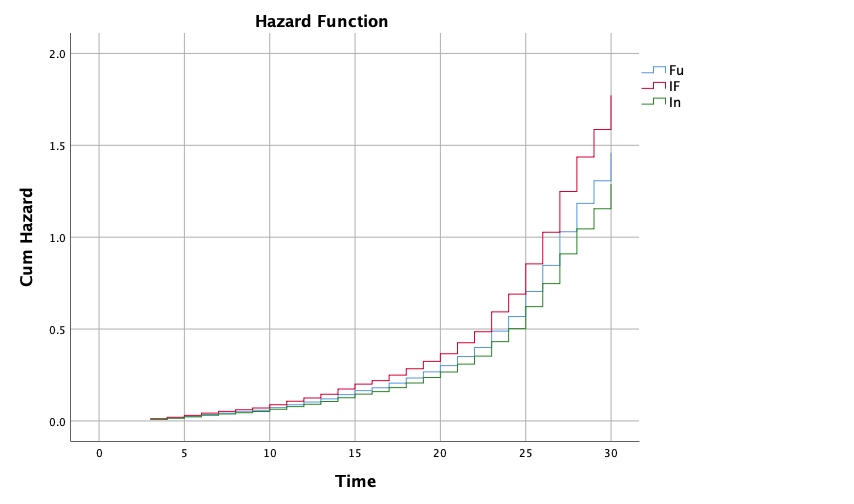


**Fig S1. Hazard function of honey bee on diets varying in pesticide content** [30 ppm fungicide propiconazole (Fu), 2 ppm insecticide chlorantraniliprole (In), 30 ppm propiconazole + 2 ppm chlorantraniliprole (IF)]. (Cox regression model).
