# Supplementary material for: Increase in longevity and amelioration of pesticide toxicity by natural levels of dietary phytochemicals in the honey bee, Apis mellifera
Source: PLoS One. 2020 Dec 9;15(12):e0243364. doi: 10.1371/journal.pone.0243364 (PMC7725320; doi:10.1371/journal.pone.0243364)
Supplement: S1 Table — (DOCX) [file pone.0243364.s002.docx]

**S1 Table.** Naturally occurring concentrations of quercetin and *p*-coumaric acid in bee products from different botanical origin.

|  | Min of mean (μM) | Average of mean (μM) | Max of mean (μM) |
| --- | --- | --- | --- |
| ***p*-Coumaric acid** |  |  |  |
| **Honey** |  |  |  |
| Acacia | 0.7 | 1.6 | 2.7 |
| Acacia tortilis | 0.1 | 0.3 | 0.5 |
| Basil | 0.0 | 0.0 | 0.0 |
| Bell heather | 2.1 | 2.1 | 2.1 |
| Black mangrove | 9.5 | 9.5 | 9.5 |
| Buckwheat | 0.0 | 0.0 | 0.0 |
| Chestnut | 1.9 | 13.0 | 30.5 |
| Christmas vine | 1.6 | 1.6 | 1.6 |
| Citrus | 1.6 | 15.2 | 25.5 |
| Cotton | 4.4 | 4.4 | 4.4 |
| Echium plantagineum | 2.0 | 2.0 | 2.0 |
| Eucalyptus | 3.6 | 12.2 | 20.7 |
| Fior di Sulla | 0.3 | 0.3 | 0.3 |
| Ghaf | 0.1 | 0.1 | 0.1 |
| Goldenrod | 0.0 | 0.0 | 0.0 |
| Heather | 0.2 | 5.8 | 11.4 |
| Honeydew | 1,914.1 | 1,914.1 | 1,914.1 |
| Jelly bush | 82.9 | 82.9 | 82.9 |
| Lavender | 1.8 | 3.2 | 4.7 |
| Lemon | 0.2 | 14.8 | 29.3 |
| Leptospermum scoparium | 0.1 | 0.1 | 0.1 |
| Lime | 6.2 | 7.4 | 8.6 |
| Linden | 0.7 | 0.7 | 0.7 |
| Linen vine | 6.6 | 6.6 | 6.6 |
| Ling heather | 20.7 | 20.7 | 20.7 |
| Manuka | 66.4 | 66.4 | 66.4 |
| Morning glory | 3.9 | 3.9 | 3.9 |
| Oak | 4.2 | 4.2 | 4.2 |
| Oilseed rape | 0.2 | 0.2 | 0.2 |
| Orange | 0.2 | 10.1 | 20.1 |
| Pine | 1.2 | 1.2 | 1.2 |
| Polyfloral | 0.2 | 41.8 | 237.6 |
| Rapeseed | 2.9 | 2.9 | 2.9 |
| Rosemary | 0.5 | 0.8 | 1.1 |
| Rosmary | 170.7 | 170.7 | 170.7 |
| Singing bean | 4.5 | 4.5 | 4.5 |
| Sulla | 17.5 | 17.5 | 17.5 |
| Sunflower | 0.5 | 2.9 | 5.3 |
| Ziziphus spina-csisti | 0.0 | 0.2 | 0.4 |
| **Pollen** |  |  |  |
| Camellia sinensis | 2,499.3 | 2,499.3 | 2,499.3 |
| Polyfloral | 4.9 | 78.9 | 153.0 |
| Schisandra chinensis | 66.2 | 66.2 | 66.2 |
| **Propolis** |  |  |  |
| Green propolis (Baccharis dracunculifolia) | 69,492.2 | 69,492.2 | 69,492.2 |
| Polyfloral | 0.0 | 9,824.1 | 55,573.4 |
| **Quercetin** |  |  |  |
| **Honey** |  |  |  |
| Acacia | 0.8 | 1.7 | 2.6 |
| Basil | 0.6 | 0.6 | 0.6 |
| Black mangrove | 1.0 | 1.0 | 1.0 |
| Buckwheat | 0.0 | 1.0 | 2.0 |
| Cherry | 3.1 | 3.1 | 3.1 |
| Chesnut | 3.7 | 3.7 | 3.7 |
| Chestnut | 5.0 | 5.0 | 5.0 |
| Christmas vine | 0.8 | 0.8 | 0.8 |
| Citrus | 1.0 | 9.5 | 19.9 |
| Clover | 6.6 | 6.6 | 6.6 |
| Cotton | 0.5 | 9.7 | 18.9 |
| Dandelion | 1.7 | 1.7 | 1.7 |
| Eucalyptus | 3.5 | 8.6 | 18.2 |
| Goldenrod | 0.9 | 0.9 | 0.9 |
| Heather | 0.0 | 0.7 | 1.3 |
| Honeydew | 29.8 | 29.8 | 29.8 |
| Jelly bush | 4.6 | 4.6 | 4.6 |
| Lavender | 0.0 | 1.0 | 2.2 |
| Lemon | 0.1 | 6.5 | 12.9 |
| Linden | 7.6 | 7.6 | 7.6 |
| Linen vine | 1.3 | 1.3 | 1.3 |
| Manuka | 14.2 | 14.2 | 14.2 |
| Maple | 2.2 | 2.2 | 2.2 |
| Melon | 1.4 | 1.4 | 1.4 |
| Morning glory | 0.8 | 0.8 | 0.8 |
| Oak | 1.3 | 1.3 | 1.3 |
| Oilseed rape | 0.1 | 0.1 | 0.1 |
| Orange | 0.3 | 2.4 | 6.3 |
| Orange blossom | 0.2 | 0.2 | 0.2 |
| Phacelia | 1.3 | 1.3 | 1.3 |
| Pine | 0.4 | 0.6 | 0.9 |
| Polyfloral | 0.1 | 7.3 | 24.3 |
| Pumpkin | 1.6 | 1.6 | 1.6 |
| Rape seed | 2.2 | 2.2 | 2.2 |
| Rapeseed | 2.3 | 2.3 | 2.3 |
| Raspberry | 1.3 | 1.3 | 1.3 |
| Rhododendron | 0.7 | 0.7 | 0.7 |
| Rosemary | 0.4 | 1.3 | 2.0 |
| Rosmary | 16.5 | 16.5 | 16.5 |
| Sage | 0.9 | 0.9 | 0.9 |
| Singing bean | 3.2 | 3.2 | 3.2 |
| Sulla | 3.2 | 3.2 | 3.2 |
| Sunflower | 0.7 | 2.7 | 6.6 |
| Thyme | 0.0 | 65.8 | 228.3 |
| **Pollen** |  |  |  |
| Cystus incanus | 3,250.0 | 3,250.0 | 3,250.0 |
| Polyfloral | 5.8 | 107.4 | 218.4 |
| Schisandra chinensis | 2,382.0 | 2,382.0 | 2,382.0 |
| **Propolis** |  |  |  |
| Polyfloral | 0.0 | 3,519.9 | 27,193.6 |
| Red propolis(Dalbergia ecastophyllum) | 8,768.0 | 8,768.0 | 8,768.0 |
| **Royal jelly** |  |  |  |
| Polyfloral | 0.7 | 0.7 | 0.7 |
