# Supplementary material for: Increase in longevity and amelioration of pesticide toxicity by natural levels of dietary phytochemicals in the honey bee, Apis mellifera
Source: PLoS One. 2020 Dec 9;15(12):e0243364. doi: 10.1371/journal.pone.0243364 (PMC7725320; doi:10.1371/journal.pone.0243364)
Supplement: S2 Table — (DOCX) [file pone.0243364.s003.docx]

**S2 Table.** Naturally occurring concentrations of quercetin and *p*-coumaric acid in bee products from different geographic origin.

|  | Min of mean (μM) | Average of mean (μM) | Max of mean (μM) |
| --- | --- | --- | --- |
| *p*-Coumaric acid |  |  |  |
| Honey |  |  |  |
| Australia | 82.9 | 82.9 | 82.9 |
| Brazil | 25.5 | 131.5 | 237.6 |
| Europe | 0.2 | 4.9 | 20.7 |
| European | 0.2 | 3.0 | 6.2 |
| Germany | 0.2 | 0.2 | 0.2 |
| Italy | 9.3 | 19.6 | 30.5 |
| New Zealand | 0.1 | 33.3 | 66.4 |
| Turkey | 0.5 | 2.9 | 7.4 |
| Cuba | 1.6 | 5.2 | 9.5 |
| United Arab Emirates | 0.1 | 0.2 | 0.3 |
| Oman | 0.0 | 0.1 | 0.2 |
| Yemen | 0.2 | 0.4 | 0.5 |
| Omani | 0.2 | 0.2 | 0.2 |
| Pakistan | 0.1 | 0.1 | 0.1 |
| Kashmir | 0.1 | 0.1 | 0.1 |
| Kingdom of Saudi Arabia | 0.8 | 0.8 | 0.8 |
| Spanish | 20.1 | 389.2 | 1,914.1 |
| Serbia | 0.0 | 0.3 | 0.7 |
| pollen |  |  |  |
| Anzer (Turkey) | 4.9 | 4.9 | 4.9 |
| China | 66.2 | 66.2 | 66.2 |
| Spain | 153.0 | 153.0 | 153.0 |
| Taiwan | 2,499.3 | 2,499.3 | 2,499.3 |
| Propolis |  |  |  |
| Anatolia(Turkey) | 230.5 | 230.5 | 230.5 |
| Argentina | 19,399.0 | 19,399.0 | 19,399.0 |
| Balkans(Croatia, Bosnia, Herecegovina, and Macedonia) | 239.6 | 239.6 | 239.6 |
| Brazil | 162.4 | 32,544.8 | 69,492.2 |
| Croatia | 0.0 | 0.0 | 0.0 |
| East Andalusia (Southern Spain) | 34.3 | 34.3 | 34.3 |
| Iraq | 808.3 | 808.3 | 808.3 |
| Italy | 55,573.4 | 55,573.4 | 55,573.4 |
| Korea | 2,844.7 | 2,844.7 | 2,844.7 |
| Polinago, Italy | 2,481.0 | 2,481.0 | 2,481.0 |
| Turkey | 8,136.1 | 8,136.1 | 8,136.1 |
| Quercetin |  |  |  |
| Honey |  |  |  |
| Australia | 4.6 | 11.4 | 18.2 |
| Brazil | 14.4 | 19.3 | 24.3 |
| China | 10.6 | 10.6 | 10.6 |
| Croatia | 0.8 | 0.9 | 0.9 |
| Egypt | 6.6 | 15.1 | 19.9 |
| European | 1.0 | 3.5 | 8.3 |
| Germany | 2.2 | 2.2 | 2.2 |
| Italy | 0.5 | 2.6 | 5.0 |
| n/a | 0.0 | 1.2 | 4.5 |
| New Zealand | 14.2 | 14.2 | 14.2 |
| Turkey | 0.1 | 0.5 | 1.3 |
| Cuba | 0.8 | 1.4 | 3.2 |
| Austria | 0.1 | 1.3 | 2.2 |
| Greece | 0.9 | 1.0 | 1.0 |
| Sicily | 0.3 | 0.3 | 0.3 |
| Kefallonia, Greece | 15.7 | 88.5 | 149.9 |
| Irakleio, Greece | 0.0 | 3.5 | 10.6 |
| Hania, Greece | 4.2 | 105.8 | 228.3 |
| Lakonia, Greece | 23.3 | 90.4 | 227.1 |
| Poland | 1.3 | 4.5 | 7.6 |
| Spanish | 6.3 | 14.8 | 29.8 |
| Serbia | 0.0 | 0.4 | 0.9 |
| Bosnia–Herzegovina | 5.2 | 5.2 | 5.2 |
| Pollen |  |  |  |
| Anzer (Turkey) | 5.8 | 5.8 | 5.8 |
| China | 2,382.0 | 2,382.0 | 2,382.0 |
| Croatia | 3,250.0 | 3,250.0 | 3,250.0 |
| Spain | 218.4 | 218.4 | 218.4 |
| Viannos, Greece | 98.1 | 98.1 | 98.1 |
| Propolis |  |  |  |
| Argentina | 0.0 | 0.0 | 0.0 |
| Brazil | 3,738.8 | 6,253.4 | 8,768.0 |
| China | 234.6 | 1,357.3 | 2,746.2 |
| Croatia | 286.8 | 286.8 | 286.8 |
| Iraq | 21.4 | 21.4 | 21.4 |
| Italy | 507.9 | 13,850.7 | 27,193.6 |
| Macedonia | 391.4 | 391.4 | 391.4 |
| n/a | 0.3 | 2.7 | 6.2 |
| Polinago, Italy | 562.5 | 562.5 | 562.5 |
| Turkey | 21,603.6 | 21,603.6 | 21,603.6 |
| Ukraine | 95.6 | 95.6 | 95.6 |
| Royal jelly |  |  |  |
| n/a | 0.7 | 0.7 | 0.7 |
